# TLR-4 Signalling Accelerates Colon Cancer Cell Adhesion via NF-κB Mediated Transcriptional Up-Regulation of Nox-1

**DOI:** 10.1371/journal.pone.0044176

**Published:** 2012-10-11

**Authors:** D. Peter O'Leary, Lavinia Bhatt, John F. Woolley, David R. Gough, Jiang H. Wang, Thomas G. Cotter, H. Paul Redmond

**Affiliations:** 1 Department of Academic Surgery, Cork University Hospital, Cork, Ireland; 2 Tumour Biology Laboratory, Department of Biochemistry, University College Cork, Cork, Ireland; Virginia Commonwealth University, United States of America

## Abstract

Surgery induced inflammation is a potent promoter of tumour recurrence and metastasis in colorectal cancer. The recently discovered family of Nox enzymes represent a major source of endogenous reactive oxygen species (ROS) and are now heavily implicated in tumour cell metastasis. Interestingly, Nox enzymes can be ‘purposefully’ activated by inflammatory cytokines and growth factors which are present in abundance in the peri-operative window. As colon cancer cells express Nox enzymes and Toll-like receptor 4 (TLR-4), we hypothesised that LPS may potentiate the ability of colon cancer cells to metastasise via Nox enzyme mediated redox signalling. In support of this hypothesis, this paper demonstrates that LPS induces a significant, transient increase of endogenous ROS in SW480, SW620 and CT-26 colon cancer cells. This increase in LPS-induced ROS activity is completely abrogated by a Nox inhibitor, diphenyleneiodonium (DPI), Nox1 siRNA and an NF-κB inhibitor, Dihydrochloride. A significant increase in Nox1 and Nox2 protein expression occurs following LPS treatment. Inhibition of NF-κB also attenuates the increase of Nox1 and Nox2 protein expression. The sub-cellular location of LPS-induced ROS generation lies mainly in the endoplasmic reticulum. LPS activates the PI3K/Akt pathway via Nox generated ROS and this signal is inhibited by DPI. This LPS activated Nox mechanism facilitates a significant increase in SW480 colon cancer cell adhesion to collagen I, which is inhibited by DPI, Nox1 siRNA and a PI3K inhibitor. Altogether, these data suggest that the LPS-Nox1 redox signalling axis plays a crucial role in facilitation of colon cancer cell adhesion, thus increasing the metastatic potential of colon cancer cells. Nox1 may represent a valuable target in which to prevent colon cancer metastasis.

## Introduction

Colorectal cancer is the second most common cause of cancer related mortality in the western world [1]. Surgical resection remains the mainstay of curative treatment for colon cancer. However, approximately 25 to 35% of node positive patients will develop either local recurrence or distant metastasis within five years post-operatively [2]. This phenomenon is due to the release of inflammatory mediators in response to surgical trauma, which potentiate the metastatic ability of circulating tumour cells and residual tumours cells. The effects of this phenomenon manifests in a significant reduction in disease free and overall survival for colorectal cancer patients.

Tumour cell adherence is an essential step of the metastatic cascade. Recent evidence has demonstrated how exogenous surgery-induced reactive oxygen species (ROS) enhance the ability of circulating tumour cells to adhere to the endothelial lining by creating intercellular gaps, allowing tumour cells to adhere preferentially to the exposed extra-cellular matrix. These destructive, cytotoxic effects of ROS occur at high levels. However, at low levels, endogenous ROS can promote cell survival through regulation of redox sensitive survival pathways such as PI3K/Akt, which has been heavily implicated in facilitating tumour cell metastasis.

Nox enzymes are a major source of endogenous ROS generation in response to inflammatory mediators such as cytokines, growth factors and hypoxic conditions, all of which are elevated in response to surgical trauma [3]–[4]. Nox enzymes consist of a family of 7 enzymes, Nox1–5 and Duox1,2 [5]. Interestingly, expression of Nox enzymes in cancer cells has recently been described and Nox-derived ROS are now known to facilitate the metastatic process in cancer cells including colon, melanoma, pancreatic and gastric cancer cells [6]–[8]. Recent evidence suggests that the signalling effects of Nox-derived ROS is context dependent, as they not only confer pro-inflammatory effects but also play a role within the cellular anti-inflammatory defence mechanism [9].

Lipopolysaccharide (LPS) or endotoxin is a potent trigger of host inflammatory responses in the peri-operative window. LPS is a gram negative bacterial antigen that translocates across the bowel wall following major surgery or during a septic episode, resulting in an endotoxaemia [10]. Recognition of LPS by Toll-like Receptor-4 (TLR4) induces innate immunity via an intra-cellular signalling cascade in a MyD88 dependent or independent manner. Both in vitro and in vivo studies now implicate LPS induced TLR-4 signalling as a trigger of every facet of the metastatic cascade including adhesion [11]–[12]. Also, TLR-4 expression in colon cancer cells is associated with an increased risk of formation of liver metastasis in colon cancer patients and confers a worse prognosis [13]–[15].

As recent evidence suggests, successful tumour cell metastasis is promoted by the destructive effects of exogenous ROS. We hypothesised that endogenous non-toxic levels of ROS can also play a major role in orchestrating tumour cell metastasis. Herein, we demonstrate how an LPS-Nox1 signalling axis gives rise to a significant increase in the adhesive ability of colon cancer cells. LPS activation of Nox activity occurs in a NF-κB dependent manner which results in a transient increase of intracellular ROS. This transient rise of intracellular ROS causes phosphorylation of redox sensitive Akt. Altogether, these data suggest that the LPS-Nox1 redox signalling axis plays a crucial role in facilitation of colon cancer cell adhesion, thus increasing the metastatic potential of colon cancer cells.

## Materials and Methods

### Cell Culture

The human colon cancer cell lines, SW480, SW-620 and CT-26 were obtained from the American Type Culture Collection (Manassas, VA). Cells were maintained in a sub-confluent state using RPMI (Roswell Park Memorial Institute) culture medium supplemented with 10% fetal calf serum, 1% penicillin/streptomycin, and 4 mmol/L of L-Glutamine all from Sigma Aldrich, Dublin, Ireland. Cells were incubated at 37°C in a humidified incubator with 5% CO_2_. Cells were plated overnight prior to LPS treatment to allow attachment.

### Antibodies and Reagents

LPS derived *E.coli* strain 055:B5 was purchased from Sigma–Aldrich. In this study the following antibodies were used - Nox1, p22phox, p47phox (Santa-cruz Biotechnology, Santa-Cruz, CA, USA), Nox2 (Upstate, Milton Keynes, UK), p-Akt(Cell Signaling), IκB-α, p-IκB-α(Cell Signalling), GAPDH(Advanced Immunochemicals, Long Beach, CA, USA). IKK inhibitor (diHydrochloride) was purchased from Sigma and PI3K inhibitor (LY294002) was purchased from EMD Chemicals (San Diego, CA, USA). Targeted knockdown of Nox1 was carried out using siRNA. Two different constructs were utilised (Ambion, #s25726, s25727).

### Flow cytometry

5×10^4^ cells per well were plated overnight in a 24 well plate. Cells were treated with LPS at a concentration of 1 ug/ml for 0,10,20,30,40,50,60 minutes and 24 hours. ROS production following LPS treatment was monitored using 2′,7′ dichlorodihydrofluorescin diacetate (DCF) (Molecular Probes, Leidin, Netherlands). Cells were trypsinised, centrifuged for 5 minutes at 1,000 rpm and then collected. DCF was then added at a final concentration of 50 µM and samples were incubated for 15 minutes at 37°C before analysis on a FACScan flow cytometer (Becton Dickinson, Oxford, UK). Dichlorofluorescin (DCF) fluorescence, is generated when DCF interacts with ROS. ROS was thus measured at fluorescence channel 1 (FL-1) (530 nm) with excitation at 488 nm. CellQuest software (Becton Dickinson) was used for data analysis.

### Western Blotting

Western Blotting was performed to measure protein content of cells treated with 1 ug/ml LPS for 0,10,20,30,40,50,60 minutes and 24 hours. Cells were then trysinised and centrifuged for collection. Whole cell extracts were then obtained. Cell pellets were washed with ice cold PBS and then resuspended in cell lysis buffer 50 mM Tris–HCl pH 7.4, 150 mMNaCl, 1 mMNa3VO4, 1 mMNaF, 1 mMEGTA,1% Nonidet P-40, 0.25% sodium deoxycholate containing protease inhibitors (Roche Diagnostics, Lewes, UK) and 0.2 mM 4-(2-aminoethyl) benzenesulfonyl fluoride hydrochloride] and incubated on ice for 20 min. All debris was removed by centrifugation at 4°C and protein concentration was quantified using the Bio-Rad protein assay (Bio-Rad, Hemel Hempstead, UK) using bovine serum albumin as a standard. Equivalent amounts of protein were resolved using denaturing sodium dodecyl sulphate–polyacrylamide gel electrophoresis, followed by transfer to nitrocellulose membranes (Schleicher & Schuell, Whatman, Dassel, Germany). Membranes were blocked with 5% (w/v) non-fat dry milk in Tris-buffered saline/0.1% Tween-20 for 1 h at room temperature. They were incubated at 4°C overnight with the appropriate dilution of primary antibody (1∶1000 unless otherwise stated). After 4×5-minute washes with Tris-buffered saline/0.1% Tween-20, blots were incubated with the corresponding peroxidase conjugated secondary antibody (dilution 1∶1000) for 1 hr. They were then washed again and developed with enhanced chemiluminescence reagent (Amersham Biosciences, Buckinghamshire, UK). Probing for GAPDH (1∶5000) was used to determine equal loading of protein.

### Immunofluorescence and Microscopy

In order to localise ROS generated on treatment with LPS in SW480 cells, cells were cultured for 48 hours before the experiment in glass bottomed dishes (35 mm Petri-dishes with 14 mm microwells; MatTek Corporation, Ashland, MA, USA). Cells used for live imaging were incubated in 50 µM DCF and 1 µM ER tracker dye (Molecular probes, Leiden, the Netherlands) for 1 hour at 37°C. One micro-molar LPS was added to the cells containing the dyes 30 minutes before imaging. Where stated, cells were treated with 10 µM DPI with DCF and ER tracker dyes for 1 hour at 37°C and 1 µM LPS was added to the cells 30 minutes before imaging. After this incubation with the dyes, cells were rinsed and imaged in either growth medium containing LPS or LPS and DPI or growth medium alone. Concentrations for DPI and LPS were maintained in the growth medium wash and while cell imaging. SW480 cells were also stained with 8 µM Menadione for 1 hr together with 10 µM MitoPY and 1 µM mitotracker deep red (Invitrogen). Menadione was used as a positive control. Additionally, cells stained with MitoPY were treated with LPS 1 µg/ml prior to imaging. SW480 cells were imaged with a multiphoton laser scanning microscope Flouview1000 MPE (Mason Technology, Dublin, Ireland) with an Infrared red Ti:Sapphire Laser that is mode locked. Images were acquired and visualised using an XLPLN 25×WMP water immersion objective (1.05 numerical aperture: Olympus Optical GmbH, Hamburg, Germany) and images were stored with an Olympus flouview1000 software (Mason Technology, Dublin, Ireland). During acquisitions, detection settings were kept constant for DCF to allow direct comparison of ROS levels, where cells treated with LPS were compared to untreated or LPS and DPI treated cells. Images have been represented as a single slice from a Z-stack projection.

### Quantitative PCR

Quantitative PCR was performed on oligo-dT generated cDNA using the MJ Research Opticon 2 detection system in combination with the Quantitect SYBR Green PCR Master Mix (Qiagen, Crawley, UK). The primers for Nox1, Nox2 and β-Actin were purchased as Quantitect Primer Assays (Qiagen). The following PCR parameters were used for each primer set: denaturing at 95°C for 15 min, followed by 45 cycles of 94°C for 15 seconds, annealing temperature of 56°C for 30 seconds and extension at 72°C for 30 seconds. RNA samples were analyzed in triplicate, and Nox1 or Nox2 expression relative to β-Actin was determined via the 2^−ΔΔCt^ method.

### Adhesion assay

96 well plates were coated with 150 µl/well of 0.01% collagen I overnight at 4°C and then blocked with 1% BSA for 1 hour at 37°C before seeding cells. 5×10^4^ cells were resuspended in 100 µl of culture medium and seeded in each well. Cells were incubated at 37°C for 1 hour. Each well was then washed with PBS and then 1% crystal violet was used to stain cells for 20 minutes. Plate was washed 3 times and then left overnight to dry at room tempeture. Crystal violet staining was then dissolved in 10% acetic acid and concentration was determined by measuring absorbance at 570 nm using a spectrophotometric microplate reader.

### Statistical Analysis

Statistical analysis was performed using SPSS version 18.1 for Windows (SPSS, Dublin, Ireland). Data are given as mean± SD. Statistical significance was evaluated by Student's t-test for comparisons between groups, and analysis of variance (ANOVA). Densitometry on western blots was performed using the programme ImageJ (from http://rsb.info.nih.gov/ij/download.html). Graphs were constructed using Microsoft Excel 2010. All graph values represent mean values +/− standard deviation. A p-value <0.05 was considered statistically significant.

## Results

### LPS induced TLR-4 signalling increases intracellular ROS levels in colon cancer cells

**Figure 1:**
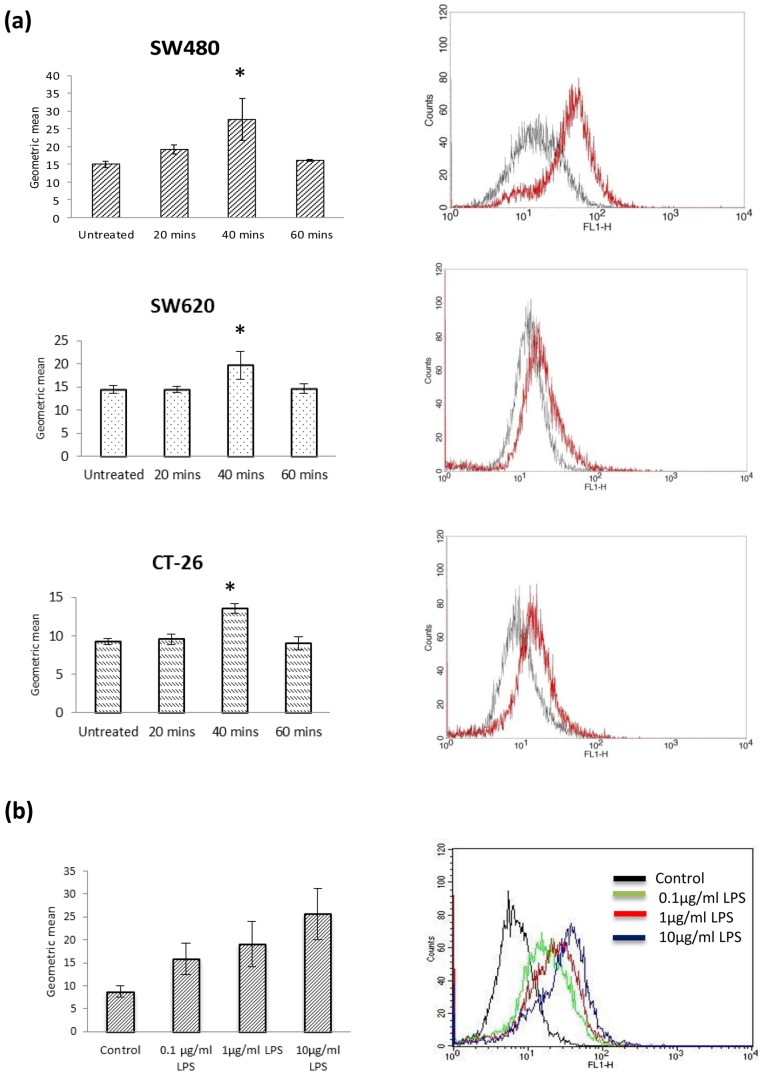
ROS are generated in response to LPS in colon cancer cells. (a) LPS induced a transient, significant rise in ROS activity in SW480, SW620, Ct-26 colon cancer cells. SW480, SW620 and CT-26 cells were treated with LPS (1 µg/ml) at twenty, forty and sixty minutes. DCF fluorescence was measured using FACScan flow cytometer and CellQuest software. These results are compiled in the above histograms. The y-axis represents cell counts and the x-axis measures the fluorescence level. A shift to the right along the x-axis represents a higher level of DCF fluorescence and thus ROS production. All histograms show a control (solid black line) with a colour line representing an increase in DCF fluorescence. The effect of LPS on ROS activity in SW480, SW620 and CT-26 colon cancer cells was quantified using CellQuest to measure the geometric means of the curves and compared to untreated controls tested on the same day, and compared in a bar chart. (b) LPS induces a dose dependent ROS burst at 40 minutes. A flow cytometry histogram demonstrates a dose dependent shift in ROS activity. (Solid black line = untreated, green = 0.1 µg/ml, red = 1 µg/ml, blue = 10 µg/ml. The dose dependent effect was quantified and compared in a bar chart. * P<0.05. These data are representative of 3 independent experiments.

SW480, Sw620 and CT-26 cells express TLR-4 and were thus chosen to examine the effect of LPS induced ROS production [16]. As determined by FACScan analysis, a transient increase of intracellular ROS levels is seen in response to LPS treatment after 40 minutes in all cell lines (Figure 1(a)). Endogenous levels of ROS are almost at control levels at 60 minutes. This data was quantified using the computer program CellQuest to measure the geometric means of the curves (Figure 1(b)). SW480 cells consistently generate higher levels of intracellular ROS in comparison to SW620 and CT-26 cells. On this basis SW480 cells are the main focus of subsequent experiments.

We next sought to examine if this LPS-induced increase in endogenous ROS was responsive to variations in the dose of LPS. We show that as the dose of LPS increases, the ROS response increases (Figure (1b)). These results indicate that SW480 cells generate endogenous ROS in response to TLR-4 signalling in a dose dependent manner in colon cancer cells.

### LPS-induced ROS generation in SW480 cells is abrogated by a Nox enzyme inhibitor

**Figure 2:**
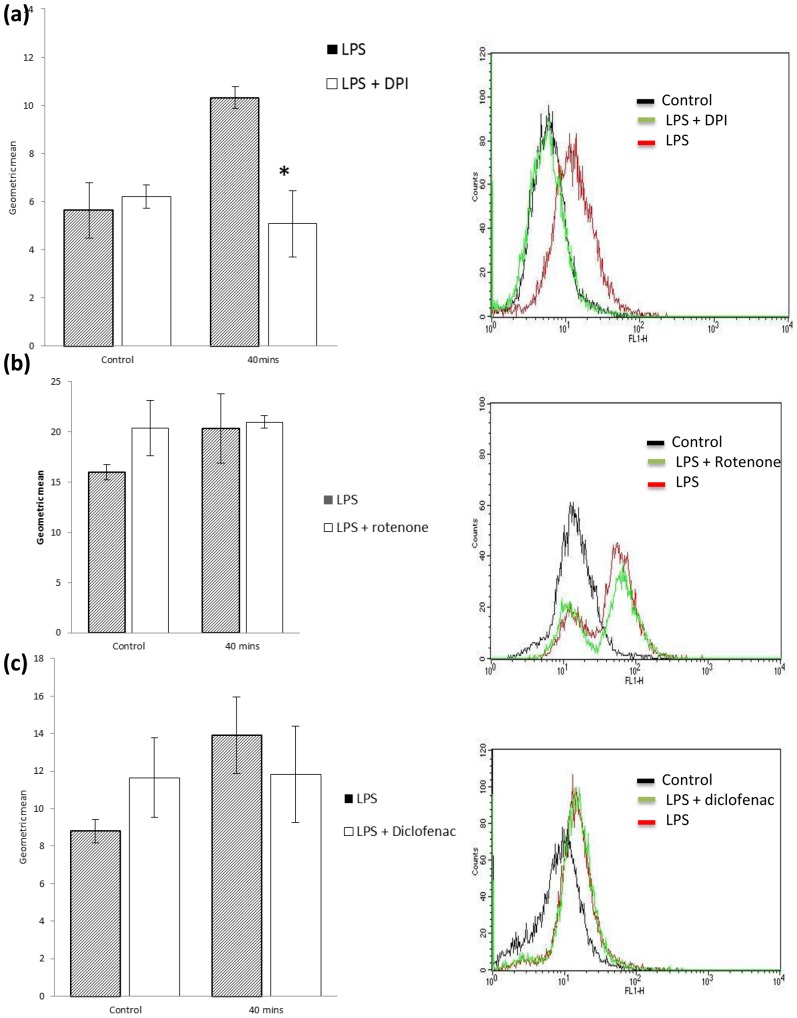
LPS induces a ROS burst via a Nox dependent mechanism in SW480 cells. (a) A significant attenuation of fluorescence was seen in samples treated with the DPI (2 µM). DCF fluorescence was measured using FACScan flow cytometer and CellQuest software. Solid black line (control). Red line (LPS treated). Green line (LPS+DPI treated). (b,c) Rotenone and diclofenac failed to inhibit LPS (1 µg/ml) induced ROS activity at 40 minutes. * P<0.05. These data are representative of 3 independent experiments.

Given that TLR-4 signalling induces a significant increase in endogenous ROS levels, we next sought to establish the intracellular source of LPS-induced ROS. LPS-induced ROS activity is known to involve TLR-4 mediated interaction with Nox4 in embryonic kidney cells [17]. However the intra-cellular source of LPS induced ROS in colon cancer cells is largely unknown. Other plausible sources of endogenous ROS generation aside from Nox enzymes include mitochondria and COX enzymes. With this in mind we used targeted inhibitors to prevent ROS generation in SW480 cells from all of these sources including a mitochondrial inhibitor (rotenone), a COX inhibitor (diclofenac) and a Nox protein inhibitor (DPI). Pre-treatment with rotenone was associated with a slight increase in ROS levels, in keeping with recent reports that rotenone can activate Nox activity [18]. Diclofenac causes no significant decrease in LPS-induced intracellular ROS levels (Figure 2 (a) and (b)). Interestingly, pre-treatment with DPI results in complete abrogation of LPS-induced ROS activity at 40 minutes (Figure 2 (c)). Taken together, these results demonstrate that Nox enzymes are the most likely intra-cellular source of ROS following TLR4 signalling in colon cancer cells.

### LPS treatment of SW480 cells increases levels of Nox1 and Nox2 expression

**Figure 3:**
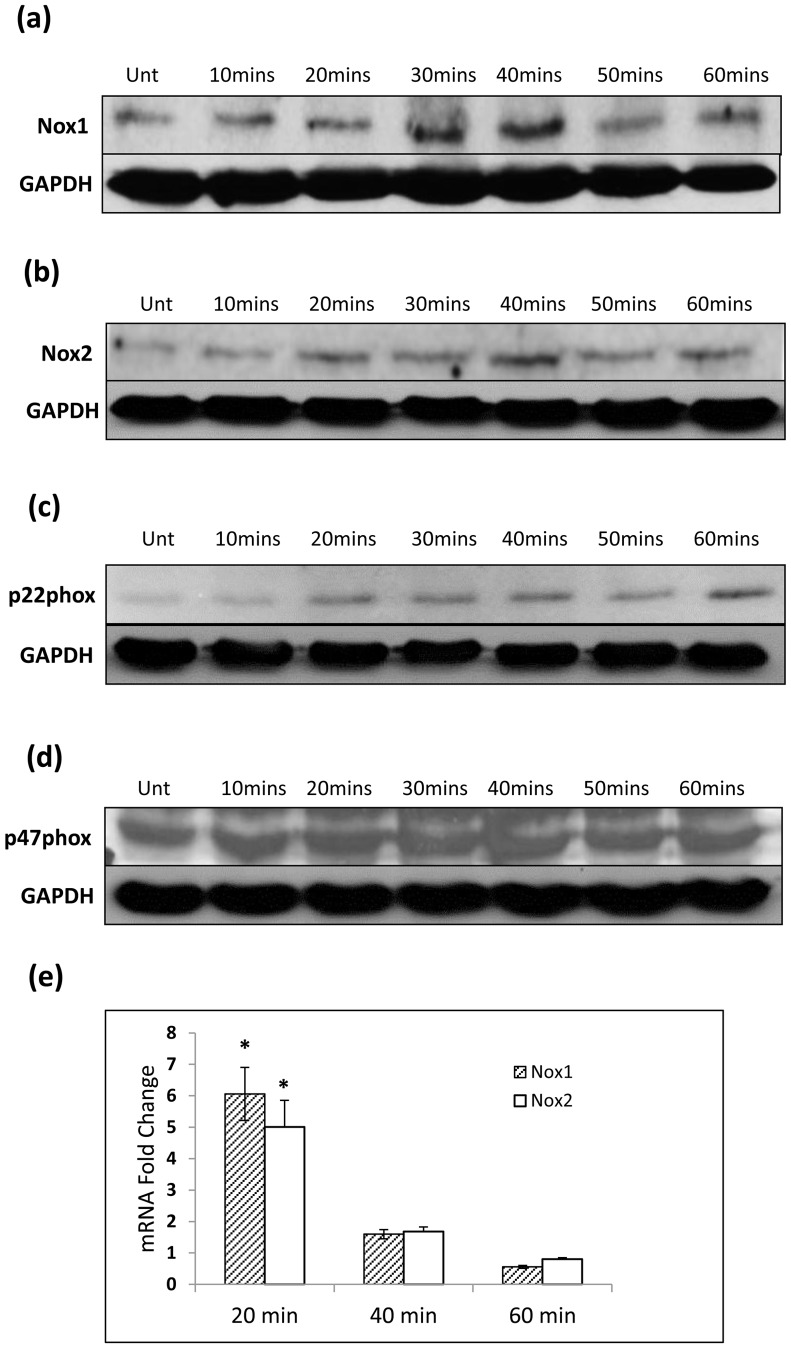
Nox1, Nox2, p22phox and p47phox are expressed in SW480 cells. (a) Using Western Blotting we show that Nox1 expression in SW480 cells increases in response to LPS (1 ug/ml). (b) Western Blotting also shows that LPS increased expression of Nox2 at 40 minutes. (c) p22phox is shown to be expressed and expression increases earlier than Nox1 and Nox2. (d) p47phox is shown to be expressed but expression is stable over the time points. (e) Quantitative PCR analysis of Nox1 and Nox2 mRNA in SW480 cells treated with LPS (1 µg/ml) over one hour. Data is represented as fold-change relative to control untreated cells hours as determined by the 2−ΔΔCt method. Results are expressed as mean±SD and are representative of three independent experiments. * P<0.05. These data are representative of 3 independent experiments.

As DPI resulted in complete abrogation of LPS-induced ROS activity, we wished to further investigate the role of Nox enzymes in LPS-induced ROS generation in SW480 cells. Western blotting was used to identify Nox enzyme expression. Nox1 as well as Nox2 were found to be expressed (Figure 3 (a),(b)). Interestingly, the level of Nox1 and Nox2 expression increases in response to LPS treatment. Nox1 protein expression increases significantly above the untreated level of expression at 40 minutes (Figure 3(a)). A similar increase is observed in the expression pattern of Nox2 (Figure 3(b)). The elevation of Nox1 and Nox2 protein expression coincides with the significant rise in LPS induced-ROS generation at 40 minutes seen on flow cytometry.

Nox enzyme activation is reliant upon assembly of individual sub-units. We therefore also looked at the effect of LPS treatment on protein expression of p22phox and p47phox (Figure 3 (c,d)). There was no change in p47phox expression, however there was an increase in p22phox expression at twenty minutes and remained sustained through to 60 minutes.

We further investigated the increase of Nox1 and Nox2 protein expression in response to LPS treatment using RT-PCR to quantify the Nox1 and Nox2 mRNA levels in response to LPS (Figure 3(e)). A dramatic 6 fold increase in Nox1 mRNA levels and a 5 fold increase in Nox2 mRNA levels compared to untreated controls occurs at 20 minutes following LPS treatment. This rise in mRNA levels is transient and there is a significant reduction by 40 minutes and 60 minutes following LPS treatment. These results suggest that LPS activates a transcription factor, most likely NF-κB, which increases Nox1 and Nox2 mRNA in response to LPS. This then facilitates an increase in Nox1 and Nox2 expression at 40 minutes which manifests with a significant increase in endogenous ROS levels in SW480 cells. In order to verify this theory, we needed to elicit the role of NF-κB in LPS induced endogenous ROS activity.

### LPS induced ROS is NF-κB dependent

**Figure 4:**
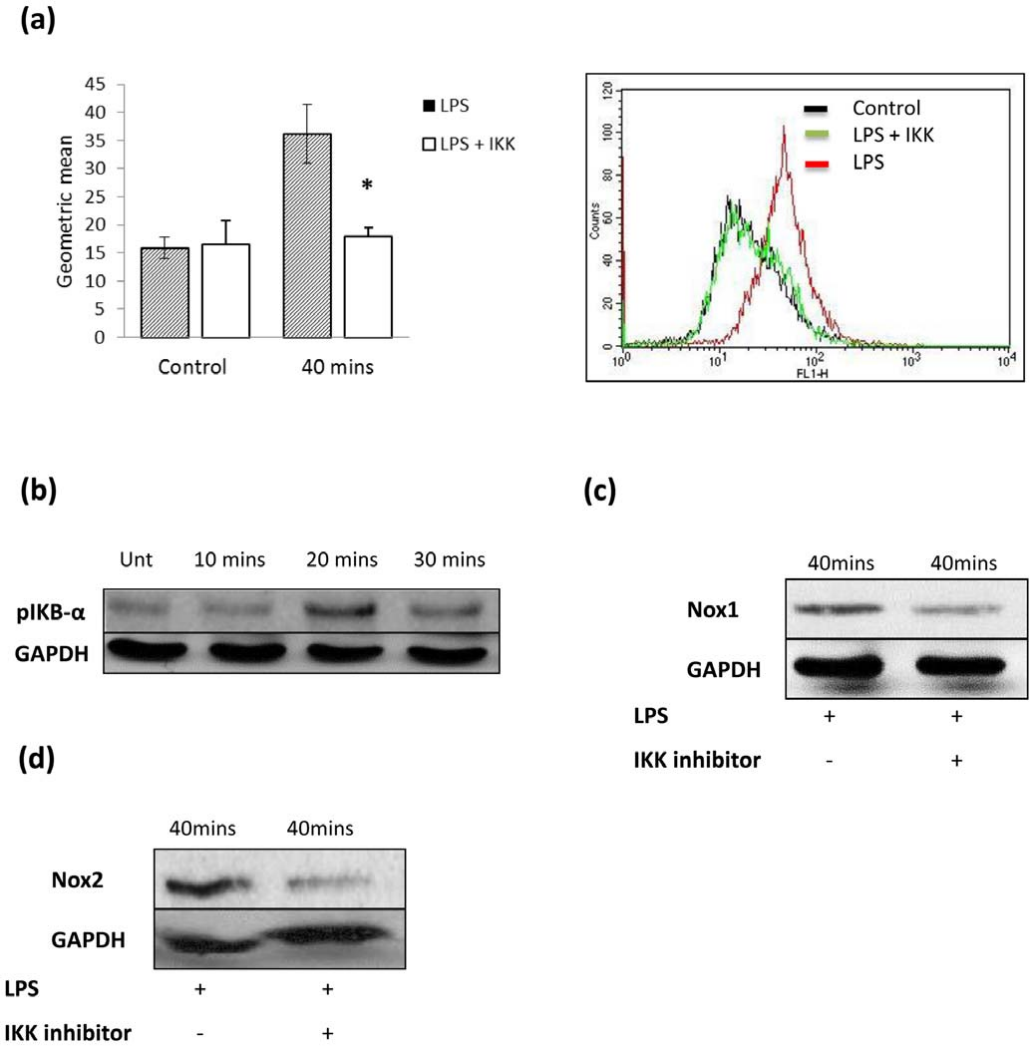
LPS induced Nox activity is NF-κB dependent. (a) Quantification of DCF fluorescence using CellQuest software demonstrates a significant reduction of LPS-induced 40 minute ROS activity in the presence of IKK inhibitor (40 µg/ml) following LPS treatment (1 µg/ml). (b) p-IκB increased in expression at 20 minutes following LPS treatment. * P<0.05. These data are representative of 3 independent experiments. (c) LPS (1 µg/ml) induced Nox1 expression increased at 40 minutes following LPS treatment, however pre-treatment with IKK inhibitor for 1 hour reduced the level of expression to untreated levels. (d) LPS induced Nox2 expression at 40 minutes is also reduced by the IKK inhibitor. * P<0.05. These data are representative of 3 independent experiments.

As there is an established link evident between LPS and NF-κB activation via the MyD88 pathway, we investigated if NF-κB played a role in LPS induced ROS activity. I-Kappa-B kinase (IKK) is an enzyme which is necessary to activate NF-κB. An IKK inhibitor was used to inhibit NF-κB activity (Figure 4 (a)). Pre-treatment with the IKK-inhibitor resulted in complete abrogation of LPS-induced ROS activity at 40 minutes. IKB-α is an inhibitory subunit of NF-κB. Phosphorylation of IKB-α is necessary for NF-κB activation. On this basis we looked at pIKB-α expression in response to LPS treatment. Interestingly, the level of pIKB-α is significantly elevated at 20 minutes following LPS treatment (Figure 4 (b)). It is likely that NF-κB activation at 20 minutes after LPS treatment allows transcription of mRNA necessary for the increase in Nox1 and Nox2 expression seen at 40 minutes.

In order to prove that NF-κB activation was required for an increase in Nox1 and Nox2 expression following LPS treatment, an NF-κB inhibitor was again used. LPS-induced Nox1 protein expression at 40 minutes was reduced by the IKK inhibitor (Figure 4 (c)). LPS-induced Nox2 protein expression was also decreased significantly at 40 minutes in the presence of the IKK inhibitor (Figure 4 (d)). Thus it appears that LPS-induced ROS generation is dependent on NF-κB activation which results in an increase of Nox1 and Nox2 expression in SW480.

### LPS-induced DCF fluorescence co-localises to the endoplasmic reticulum in SW480 cells

**Figure 5:**
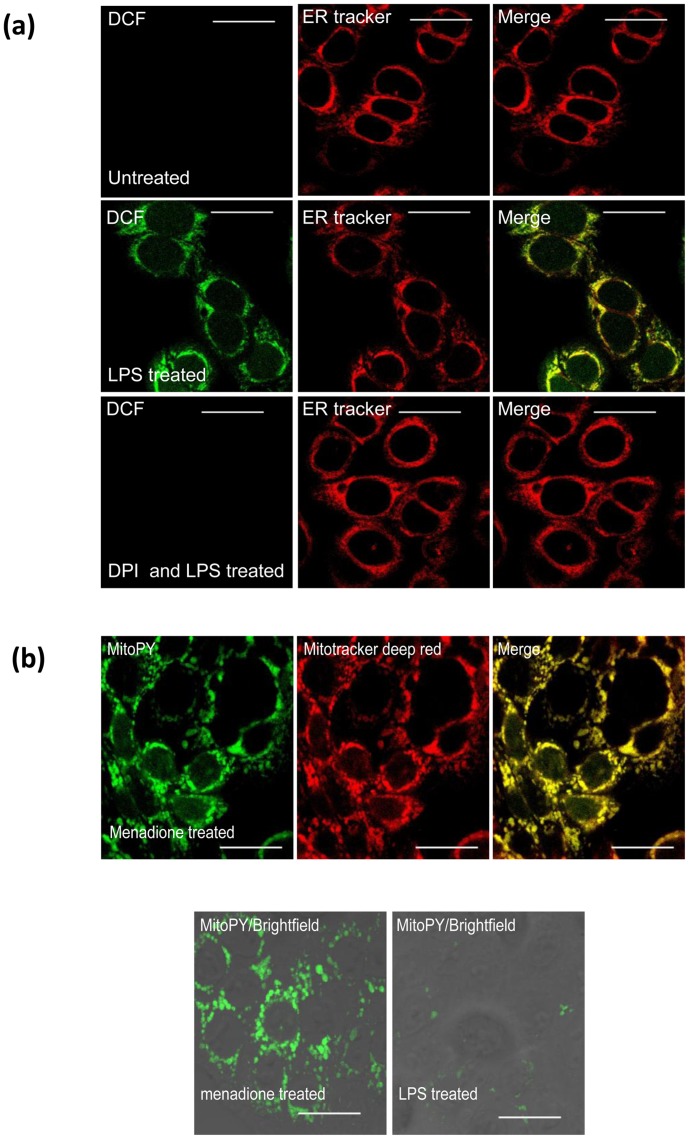
ROS generated in response to LPS appears to co-localise to the endoplasmic reticulum in SW480 cells. Cells were cultured for 48 hours in glass bottomed dishes. (a) Cells were treated with DCF and an ER tracker dye for 1 hour at 37°C with LPS (1 µg/ml) added 30 minutes before imaging with a multiphoton laser scanning microscope. (b) Cells were stained with mitotracker deep red (1 µM) and (10 µM) MitoPY, and treated with Menadione (8 µM) or LPS (1 µg/ml) for 1 hour at 37°C. The scale bar represents 20 µm.

Recent anti-oxidant therapies are more effective due to greater bioavailability and the ability to target specific ROS generating organelles such as the mitochondria. As there was no decrease in ROS levels with the use of rotenone, we questioned the subcellular compartment that was responsible for ROS generation in response to LPS. We first co-stained SW480 cells with DCF and an ER tracker dye and then examined fluorescence using multiphoton microscopy. DCF displays a distinct peri-nuclear staining pattern in SW480 cells in response to LPS treatment (Figure 5a). This pattern is very similar to what we observe with an ER tracker dye and co-localises with DCF. This suggests that the increase in ROS following LPS treatment is generated in the ER. When SW480 cells were treated with DPI, this attenuates the DCF fluorescence in the ER, thus providing further evidence that LPS-induced ROS activity is associated with Nox enzyme expression. These experiments indicate that ROS generated in response to LPS localise to the ER and together with recent evidence which shows that the TLR-4/MD2 complex signals through the ER, demonstrate that the ER plays a focal role in TLR-4 signalling.

We next wanted to examine the mitochondria as an intracellular source of LPS induced ROS. A previous study by Dickenson et al demonstrated that peroxy-yellow (MitoPY), a fluorescent probe, can effectively be used to image H_2_O_2_ within the mitochondria of live cells [19]. Here, we co-stained MitoPY with mitotracker deep red and then treated the SW480 cells with Menadione at 8 µM for 1 hour at 37°C (Figure 5b) [20]. We confirmed that MitoPY is targeted to the mitochondria and detects mitochondrial ROS when cells were treated with Menadione. We then treated SW480 cells with LPS and observed minimal levels of ROS generated in the mitochondria in comparison to the positive control where cells were treated with Menadione.

### TLR-4 induced ROS regulate metastatic signal transduction pathways in SW480 cells

**Figure 6:**
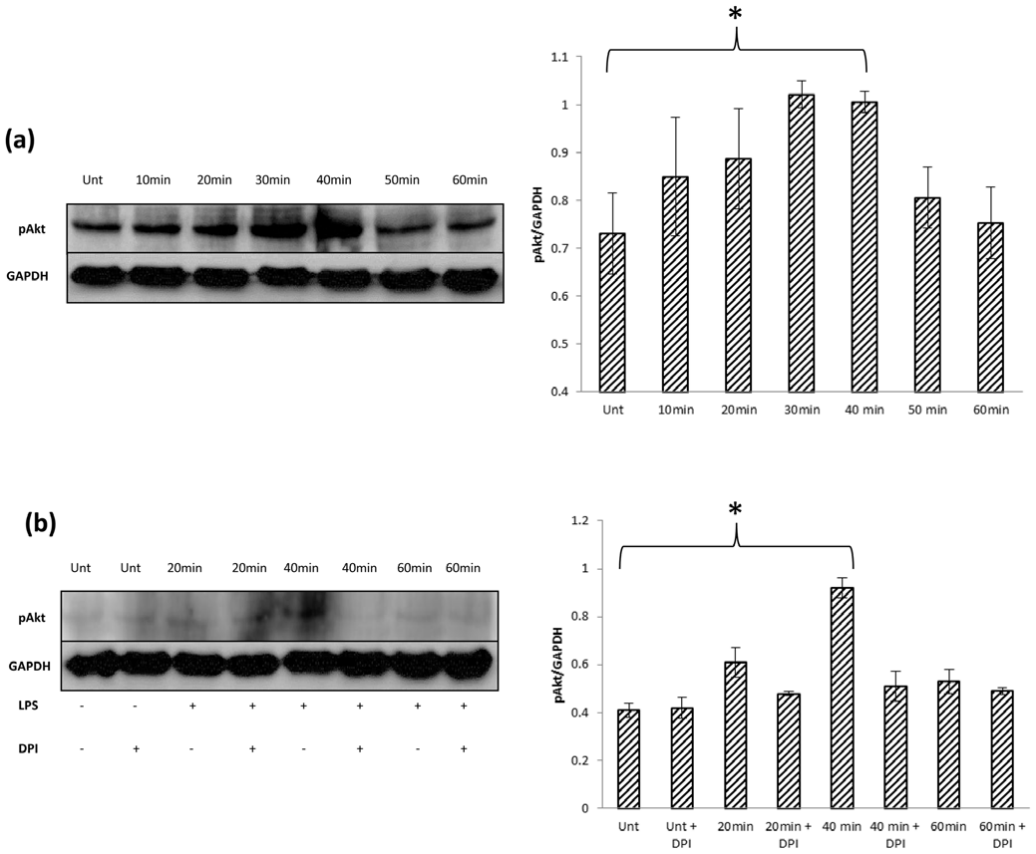
LPS regulates PI3K/Akt signalling via Nox derived ROS. (a) LPS (1 µg/ml) treatment causes a transient increase in Akt phosphorylation, maximal at 30–40 minutes. (b) pAkt expression is increased in response to LPS treatment at 40 minutes and completely inhibited by DPI(2 µM). *P<0.05. These data are representative of 3 independent experiments.

Regulation of cellular signalling pathways is one of an array of intra-cellular effects derived from TLR-4 signalling. Individual survival pathways including the PI3K/Akt pathway, which is known to facilitate tumour metastasis, can be activated by TLR-4 signalling. The inhibitory proteins of this pathway such as PTEN, are known to be redox sensitive. Having established that SW480 cells generate endogenous ROS from the ER in an NF-kB dependent manner in response to TLR-4 signalling, we thus investigated if LPS induced endogenous ROS could play a role in regulation of these redox sensitive pathways. We see an increase in pAkt at 40 minutes following LPS treatment which corresponds with the increase in LPS-induced ROS activity also seen at 40 minutes (Figure 6 (a)). pAkt expression at 40 minutes is inhibited by DPI (Figure 6 (b)). These results demonstrate how TLR-4 induced ROS activity can regulate metastatic signalling pathways such as PI3K/Akt.

### LPS induced adhesion is facilitated in a Nox dependent manner

**Figure 7:**
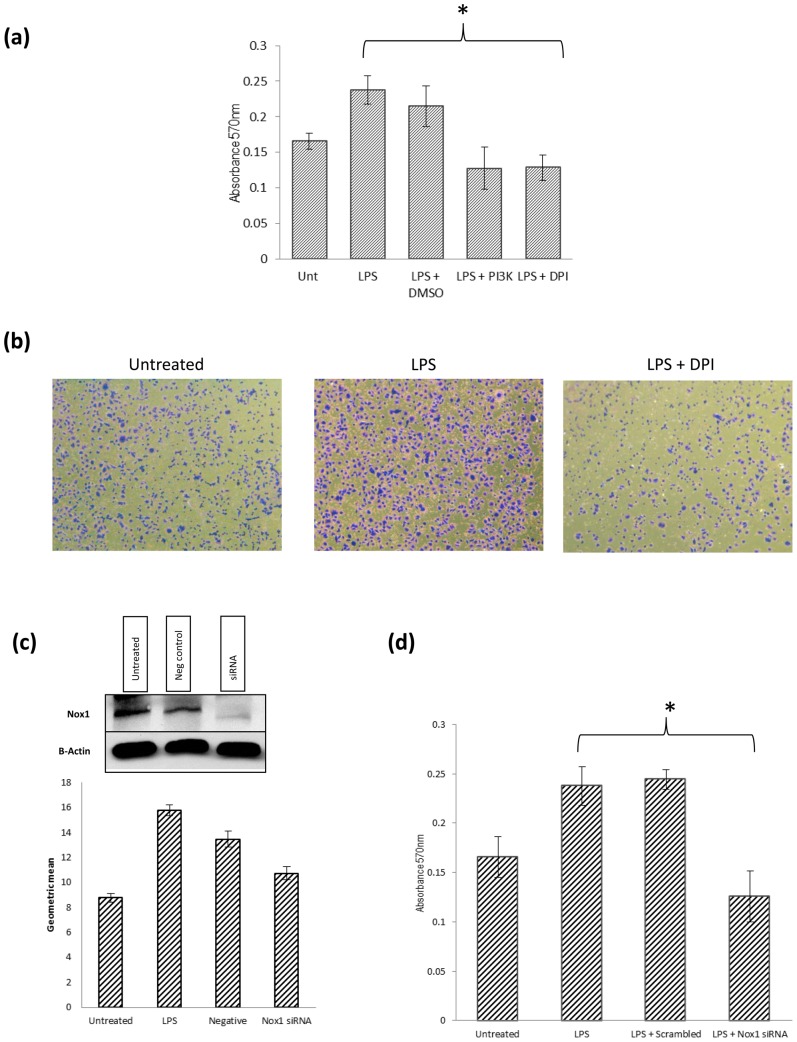
LPS accelaerates SW480 colon cancer cell adhesion via Nox regulation of PI3K/Akt signalling. (a,b) LPS treatment resulted in a significant increase in SW480 cell adhesion to collagen I after 1 hour. This was inhibited using a recognised Nox inhibitor, DPI (2 µM), and a PI3K inhibitor, LY294002(5 µM). (c) Nox1 siRNA abrogates LPS induced ROS and Nox1 knockdown is confirmed by Western Blotting. (d) The increase in SW480 colon cancer cell adhesion was completely inhibited by Nox1 siRNA. * P<0.05. These data are representative of 3 independent experiments.

Recent studies have reported that LPS can increase cancer cells adherence, an essential step for successful metastasis [21]. LPS facilitates this through up-regulation of proteins such as β1-Integrin [22]. The main signalling pathway responsible is the PI3K/Akt pathway which we have shown to regulated by LPS activation of Nox-derived ROS. Thus we wished to establish if Nox signalling facilitated LPS colon cancer cell adherence in response to LPS. A significant increase in SW480 cell adherence to Collagen I is seen at 1 hour (Figure 7 (a,b)). This increase in adherence is inhibited by DPI and a PI3K inhibitor, LY294002. This suggests that the mechanism responsible is dependent on Nox signalling. Having shown that LPS induces ROS via a Nox mechanism, we wished to investigate further which member of the Nox family was responsible for this increase in ROS activity and tumour cell adhesion. The effectiveness of the siRNA agent was tested using Flow cytometry. The second siRNA provided the best reduction and indeed caused an almost complete abrogation of LPS induced ROS (Figure 7 (c)). Thus it appears Nox1 contributes the majority of ROS and perhaps has a more functional role than Nox2 in response to LPS treatment. Western Blotting was used to confirm the effectiveness of the each siRNA transfection (Figure 7 (c)).

Nox1 siRNA treated cells were then used to investigate the role of Nox1 in colon cancer cell adherence. Interestingly, a significant reduction in tumour cell adherence was seen in the Nox1 siRNA cells following LPS treatment, indicating that Nox1 derived ROS are essential for potentiation of colon cancer cell adherence in response to LPS (Figure 7 (d)).

## Discussion

The link between systemic inflammation and promotion of tumour metastasis has been well established [23]. In this study, we demonstrate how Nox-derived ROS provide a ‘missing link’ in the understanding of how inflammation activates the signal transduction pathways responsible for tumour recurrence and metastasis. This study demonstrates that LPS regulates PI3K/Akt signalling via Nox-derived ROS in colon cancer cells. Consequently, this LPS activated redox mechanism is responsible for promoting tumour adherence, potentiating the risk of successful metastasis.

Surgical inflammation potentiates tumour cell metastasis through PI3K/Akt signalling. Previous studies have shown a significant rise in pulmonary metastasis following surgery and this effect is inhibited by a PI3K inhibitor [24]. Similarly, Hsu et al recently demonstrated that tumour cell adhesion is dependent upon PI3K signalling in response to LPS [11]. Indeed, LPS has been shown to directly activate PI3K/Akt signalling, however as our results show, Nox-derived ROS play a major role in the redox regulation of this pathway [25]–[26]. Interestingly, previous studies have shown that Nox-derived ROS are activated downstream of PI3K/Akt signalling [27]. Thus it appears that Nox-derived ROS have a role both upstream and downstream of PI3K/Akt signalling in tumour cells.

This study shows that the potentiating effects of inflammation on tumour cell metastasis are derived from endogenous ROS, predominantly generated from Nox1 in colon cancer cells. This effect is inhibited by Nox1 siRNA which may well represent a useful target to prevent effects of inflammation on promotion of tumour cell metastasis. Nox1 expression in colon cancer has been previously described and activation of Nox1 in colon cancer has been shown to be an important mediator of invadopodia formation which can facilitate cancer cell invasion [28]–[30]. Nox1 is also required for Ras-mediated VEGF expression in colon cancer cells and has been shown to be a key player in the angiogenic switch [31]. We demonstrate that NF-κB derived transcriptional up-regulation of Nox1 potentiates cancer cell adherence in response to an inflammatory stimulus.

Interestingly, the level of Nox1 expression was seen to increase corresponding with ROS production following LPS treatment. Although the levels of Nox enzyme expression was weak in untreated cells, the capacity of the Nox family to generate endogenous ROS in response to an inflammatory stimulus should not be underestimated. TNF-α, a vital cytokine produced by the inflammatory response has previously been reported to increase transcription of Nox1 and increase superoxide production in colon cancer cells after 24 hours [32].

A functional homology has been suggested to exist between Nox1 and Nox2 (gp91phox) in phagocytes [33]. Knowledge of their characteristics in non-phagocytic cells is limited. This study demonstrates that both Nox1 and Nox2 expression increases synergistically in colon cancer cells to produce ROS following LPS treatment. Nox2 expression levels were very low in the untreated group. However, they increased following LPS treatment, in tandem with Nox1 protein expression levels. Although an increase in Nox2 expression is seen following LPS treatment, the functional value of Nox2 in LPS regulation of the PI3K/Akt pathway seems to be limited as Nox1 siRNA abrogated the majority of ROS. Also Nox1 siRNA inhibited the effects of LPS on tumour cell adherence.

TLR-4 signalling activates NF-kB through the MyD88 pathway and leads to transcription of pro-inflammatory cytokines and many important components of the inflammatory response. Previously, NF-kB has provided a mechanistic link between inflammation and cancer [34]. NF-kB activity has also been shown to be regulated by Nox-derived ROS [29]. However this study demonstrates that a mechanistic link occurs via Nox-derived ROS in colon cancer cells. Generation of Nox derived ROS is dependent on NF-kB activity in colon cancer cells.

This study provides further insight into the subcellular location of TLR-4 induced ROS. An association between TLR-4/MD2 complex signalling and the ER has previously been made. Our study provides evidence of the role of the ER in TLR-4 signalling as it was observed that the ER was the subcellular location responsible for LPS-induced ROS generation which regulate redox sensitive signalling pathways. An important therapeutic implication of this finding relates to the potential use of targeted anti-oxidant therapy to counteract the redox signalling effects conferred by TLR-4 activation. Mitochondria have traditionally been attributed as the main intracellular source of ROS and are being targeted with the latest generation of anti-oxidants [35]. However, as we demonstrate by using rotenone, minimal amounts of the TLR-4 induced ROS are sequestered from mitochondria and in fact the majority are generated mainly in the endoplasmic reticulum. Targeted anti-oxidant therapy and Nox-1 inhibition are two potential methods to target the effects metastasis potentiating effects of the inflammatory insult provoked by surgery in the peri-operative window.

In summary, unravelling of the LPS-Nox1 signalling axis reveals potential redox targets that could be used to prevent successful tumour metastasis in response to inflammation.
